# A Cylindrical High-Temperature-Resistant Fiber-Optic Composite Sensor for Temperature and Pressure Measurement

**DOI:** 10.3390/s26020417

**Published:** 2026-01-08

**Authors:** Siwei Zhang, Quan Liu, Jiaqi Liu, Jiahao Guo, Ruiya Li

**Affiliations:** 1 School of Information Engineering, Wuhan University of Technology, Wuhan 430070, China; swzhang@whut.edu.cn (S.Z.); quanliu@whut.edu.cn (Q.L.); 2 School of Mechanical and Electronic Engineering, Wuhan University of Technology, Wuhan 430070, China; 325351@whut.edu.cn (J.L.); g030626@whut.edu.cn (J.G.)

**Keywords:** extrinsic Fabry–Pérot interferometer, fiber Bragg grating, high-temperature, pressure, cylindrical pressure chamber

## Abstract

This study proposes a cylindrical high-temperature-resistant fiber-optic composite sensor based on the EFPI-FBG hybrid structure for simultaneous temperature and pressure measurement, addressing the demand for high-performance monitoring in harsh environments. The sensor’s core consists of a cylindrical pressure chamber, a metal substrate, and an EFPI-FBG sensing structure fixed via resistance welding and high-temperature ceramic adhesive. The cylindrical pressure chamber converts pressure into axial deformation to modulate the EFPI cavity length, while the FBG with one end floating is exclusively used for temperature compensation, avoiding pressure interference. The EFPI cavity length exhibits a linear relationship with pressure, achieving a sensitivity of 0.171 μm/MPa and a linear correlation coefficient of 0.9986. Stable operation up to 600 °C and 20 MPa is demonstrated, with a decoupling matrix enabling accurate dual-parameter sensing.

## 1. Introduction

Energy and power systems rank among the most demanding fields for high-temperature pressure measurement, featuring intensive requirements and extreme operational conditions. Representative applications include pressure monitoring within the combustion chambers of gas turbines and aero-engines [1,2], as well as at turbine inlets and outlets [3,4]. Similarly, such measurements are critical in rocket engine combustion chambers and the inlet/outlet sections of turbopumps [5,6]. Furthermore, significant demand for high-temperature pressure sensing exists in various industrial sectors, including manufacturing, chemical processing, and metallurgical operations [7,8].

Conducting pressure measurements in high-temperature environments presents substantial challenges to the thermal resilience of both sensor materials and packaging technologies [9,10]. Based on the material of the sensing element, high-temperature pressure sensors can be categorized into silicon-based [11,12], piezoelectric-based (e.g., ceramics and quartz) [13,14], and sapphire-based types [15]. According to their operational principles, they also can be classified into piezoresistive [16], piezoelectric [17], capacitive [18], and optical [19] variants.

Fiber optic sensing technology [20], which originated from advancements in optical fiber communication [21], utilizes sensors [22,23] known for their high sensitivity, immunity to electromagnetic interference, and capability for multipoint measurement. A key advantage lies in the sensing material itself; the silicon dioxide core of optical fibers exhibits exceptional high-temperature resistance. In recent years, this technology has demonstrated significant potential for the multi-physical parameter monitoring of equipment operating under high-temperature conditions [24]. Recent advances in high-temperature fiber-optic sensors have showcased diverse strategies in material selection, structural design, and functional integration to enhance performance under extreme conditions. Li et al. [25] developed an all-silica Fabry–Pérot (FP) sensor capable of operating up to 800 °C, achieving a pressure sensitivity of 3.25 μm/MPa with minimal thermal drift (0.435 nm/°C). Its adhesive-free, batch-producible design ensures reliability under thermal cycling. Similarly, Liao et al. [26] implemented a sapphire MEMS-based FP sensor with integrated temperature compensation, enabling high-accuracy pressure sensing up to 1500 °C and a post-compensation accuracy of 0.86% F.S., making it suitable for nuclear reactor monitoring. In the pursuit of multi-parameter sensing, Zhang et al. [27] designed an all-sapphire FP sensor for simultaneous temperature and pressure measurement up to 1400 °C, maintaining a pressure sensitivity of 0.3253 μm/MPa even at the maximum temperature. Cao et al. [28] further expanded functionality with an all-rigid FP sensor that combines static pressure sensing (2.91 nm/MPa at 500 °C) and acoustic detection within a 20 Hz ~ 20 kHz band, supporting multi-modal monitoring in aerospace engines. Efforts in minimizing cross-sensitivity are exemplified by Li et al. [29], who reported an all-SiC FP sensor with a low temperature-pressure cross-sensitivity of 4.98 °C/MPa and high pressure sensitivity (174.3 nm/MPa) at 700 °C. In a different approach, Feng et al. [30] introduced an FBG (Fiber Bragg Grating) sensor incorporating a diaphragm and special-shaped bracket, which exhibited excellent linearity, repeatability, and minimal hysteresis within 50~200 °C and 0~40 MPa, highlighting the role of mechanical design in sensor stability.

The existing research demonstrates several common limitations. Most fiber-optic pressure sensors (e.g., [25,26,29,30]) operate within a relatively low pressure range (typically 0~1 MPa), which may not meet the demands of practical industrial applications such as energy and power systems requiring higher pressure monitoring. While some studies [27,28] attempt to achieve multi-parameter sensing, they often face difficulties in effectively decoupling temperature and pressure signals. The absence of integrated compensation mechanisms or dedicated reference structures can lead to notable cross-sensitivity, thereby constraining measurement accuracy in varying thermal and mechanical conditions. Certain designs relying on specialized materials (e.g., all-sapphire or all-SiC) face challenges in terms of fabrication complexity, structural robustness, or scalable production. Moreover, the use of adhesives in some sensor assemblies introduces reliability concerns under prolonged high-temperature exposure due to mismatches in the coefficient of thermal expansion. Compared to existing works, the innovations and contributions of this study are as follows:

(1) Using a cylindrical structure as the elastic body, the total elongation of the tubular elastic body under pressure is concentrated into a change in the distance between the two reflective surfaces of a segmented EFPI (Extrinsic Fabry–Pérot Interferometer). Compared with traditional hollow-core fiber-based Fabry–Perot cavities, this design achieves a significant enhancement in pressure sensitivity.
(2) By integrating an EFPI cavity for pressure sensing and an FBG with a floating end dedicated to temperature measurement, this design achieves clear functional separation. A decoupling matrix is established, enabling accurate dual-parameter measurement, which enhances measurement reliability in complex thermal-mechanical environments.
(3) Compact and robust sensor packaging for practical applications: The sensor employs a cylindrical metal-ceramic composite structure fixed with high-temperature ceramic adhesive, providing mechanical stability, ease of installation, and resistance to thermal mismatch.

The remainder of this paper is organized as follows: Section 2 describes the sensor structure design, sensing principle, and the high-temperature-resistant fabrication; Section 3 presents experimental validation and results analysis; and Section 4 provides concluding remarks.

## 2. Materials and Methods

### 2.1. Structure Design of the Composite Sensors

**Figure 1:**
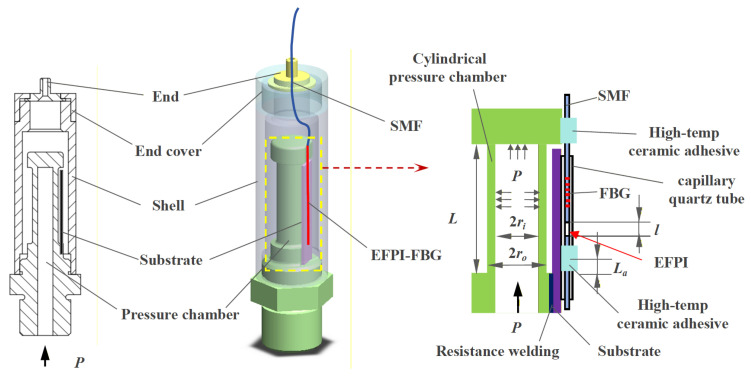
Schematic diagram of sensor structural design. (SMF: Single Mode Fiber. FBG: Fiber Bragg Grating. EFPI: Extrinsic Fabry–Pérot Interferometer).

As illustrated in Figure 1, the core pressure-sensing element of the sensor is a cylindrical pressure chamber component. A metal substrate is resistance-welded to one side of this pressure chamber. An EFPI-FBG hybrid sensing structure, secured via two-point mounting using high-temperature-resistant ceramic adhesive, is installed across the pressure chamber and metal substrate: one end is affixed to the lateral surface of the chamber’s distal end, and the other to the substrate surface.

This EFPI-FBG structure is formed by inserting a single-mode fiber (with FBG and polished end-face) into a capillary quartz tube. Notably, the opposite end of the capillary tube is pre-inserted with another polished-end single-mode fiber, and they are fusion-spliced via arc welding. This configuration creates an extrinsic Fabry–Pérot interferometer (EFPI) cavity between the two fiber end-faces.

Under increasing internal pressure, the cylindrical wall undergoes radial expansion and axial elongation, thereby increasing the EFPI cavity length. This design concentrates the axial deformation of the pressure chamber (length *L*) entirely into measurable EFPI cavity length changes, achieving significantly higher sensitivity than direct strain measurement on the cylinder wall.

The FBG remains free-floating at one end, rendering its center wavelength largely pressure-insensitive; it functions exclusively for temperature compensation. Fabricated primarily from metals, quartz, and ceramics, the sensor exhibits exceptional high-temperature compatibility.

### 2.2. Principle of Sensing

**Figure 2:**
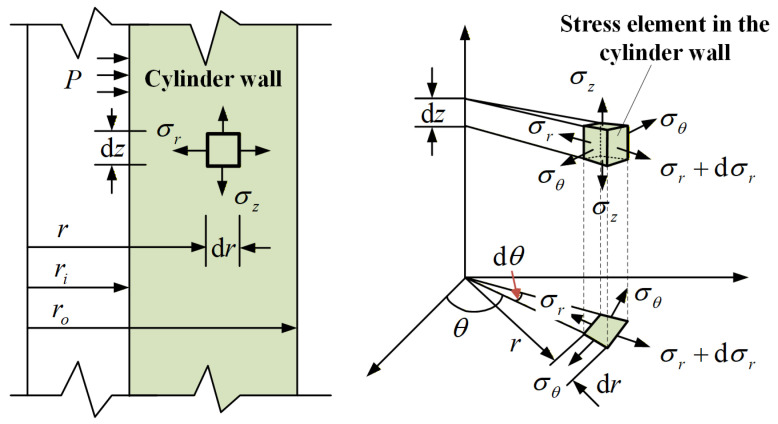
Cylinder wall stress analysis.

Under internal pressure loading on the cylindrical wall surface, the material within the cylindrical wall develops triaxial normal stresses, including radial stress $\sigma_{r}$, hoop stress $\sigma_{\theta }$, and axial stress $\sigma_{z}$, as shown in Figure 2.

Assuming that: (1) the sensor is modeled as a uniform thin-walled cylinder, with a wall thickness-to-diameter ratio assumed to be less than 1:10; (2) during pressure loading, the temperature of the air inside the EFPI cavity remains constant, based on the theory of material mechanics, by establishing static equilibrium equations, physical equations, and geometric equations, it is ultimately derived that under pressure *P*, the stresses in the cylindrical wall material can be calculated by Equation (1).

(1) $$\left\{\begin{array}{c} \sigma_{r}=\frac{r_{i}^{2}}{r_{o}^{2}-r_{i}^{2}}\left(1-\frac{r_{o}^{2}}{r^{2}}\right)P\\ \sigma_{\theta }=\frac{r_{i}^{2}}{r_{o}^{2}-r_{i}^{2}}\left(1+\frac{r_{o}^{2}}{r^{2}}\right)P\;\\ \sigma_{z}=\frac{r_{i}^{2}}{r_{o}^{2}-r_{i}^{2}}P \end{array}\right.$$

where *r_o_* denote the outer radius of the cylinder and *r_i_* its inner radius. According to the Generalized Hooke’s Law, the axial strain of the cylinder wall can be derived as Equation (2).

(2) $$\varepsilon_{z}=\frac{1}{E}\left[\sigma_{z}-\mu \left(\sigma_{r}+\sigma_{\theta }\right)\right]=\frac{Pr_{i}^{2}\left(1-2\mu \right)}{E\left(r_{o}^{2}-r_{i}^{2}\right)}$$

where E represents the Young’s modulus of the cylinder wall material and μ is Poisson’s ratio. Consequently, under applied pressure P, the cavity length variation in the EFPI can be expressed as Equation (3).

(3) $$\Delta l_{P}\approx \Delta L_{P}=\varepsilon_{z}L=\frac{PLr_{i}^{2}\left(1-2\mu \right)}{E\left(r_{o}^{2}-r_{i}^{2}\right)}$$

where $\Delta L_{P}$ represents the axial displacement of a cylinder with initial effective length L under pressure loading. In addition to pressure, variations in the EFPI cavity length may also arise from thermal expansion of the overall structure. Assuming the metal substrate and pressure chamber component share identical material properties with a coefficient of thermal expansion (CTE) $\alpha_{v}$, while the optical fiber core has a CTE $\alpha_{f}$, and denoting the distance between the two ceramic adhesive bonding points in Figure 1 as $L_{a}$, the temperature-induced cavity length change under environmental temperature variation ΔT is expressed as Equation (4).

(4) $$\Delta l_{T}=\left(\alpha_{v}-\alpha_{f}\right)L_{a}\Delta T$$

**Figure 3:**
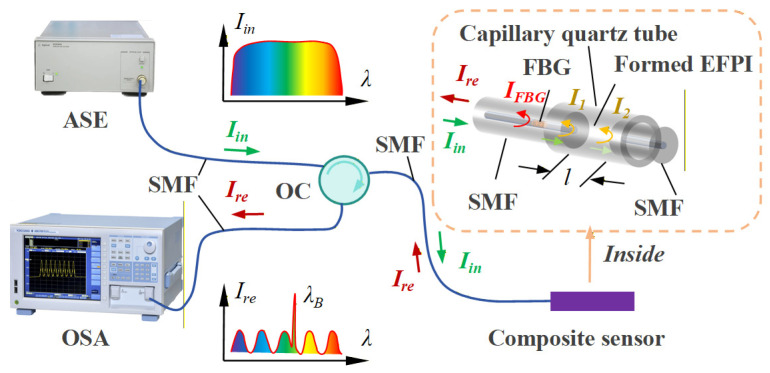
Sensing principle of EFPI-FBG. (ASE: Amplified Spontaneous Emission (light source). SMF: Single Mode Fiber. OSA: Optical Spectrum Analyzer. OC: Optical Circulator).

Based on the preceding analysis, precise sensing of ambient temperature variations via the FBG enables the detection of pressure changes through monitoring of EFPI cavity length variations. The operating principle of the EFPI-FBG hybrid sensing structure is illustrated in Figure 3.

When a broadband optical signal Iin propagates through the single-mode fiber and initially reaches the FBG, a narrowband spectrum *I*_FBG_ approximating a Gaussian distribution is reflected, while the remaining light transmits through the FBG toward the two reflective surfaces of the EFPI. Denoting the light intensities reflected from these two EFPI surfaces as *I*_1_ and *I*_2,_ respectively, interference occurs between these two beams due to the optical path difference (OPD). The FBG reflection spectrum IFBG and the EFPI reflection spectrum IEFPI can be given by Equations (5) and (6).

(5) $$I_{FBG}=I_{in}\cdot R\cdot \exp\left[-\frac{4\ln2{\left(\lambda -\lambda_{B}\right)}^{2}}{w^{2}}\right]$$

(6) $$I_{EFPI}=I_{1}+I_{2}+2\sqrt{I_{1}I_{2}}\cos\left(\frac{4\pi nl}{\lambda }+\phi_{0}\right)$$

where $I_{in}$ is Incident light intensity, *R* denotes the reflectivity of the FBG, *λ*_B_ represents the Bragg wavelength (i.e., the center wavelength of the FBG reflection spectrum), and *w* is the 3 dB bandwidth of the FBG reflection spectrum; *n* signifies the refractive index of the medium within the EFPI cavity (here, air), *l* denotes the physical cavity length of the EFPI, and $\phi_{0}$ is the initial phase of the EFPI cavity. Ultimately, the spectrum detected by the spectrometer constitutes a superposition of the FBG narrowband spectrum and the EFPI interference spectrum as shown in Equation (7).

(7) $$I_{re}=I_{EFPI}+I_{FBG}$$

By applying intensity thresholding or gradient-based detection to the composite spectrum, the peak region of the FBG reflection spectrum can be isolated. Subsequently, the center wavelength of the FBG *λ*_B_ can be determined via centroid peak detection or Gaussian fitting. The physical relationship holds as following Equation (8).

(8) $$\lambda_{B}=2n_{eff}\Lambda$$

where *n*_eff_ denotes the effective refractive index of the fiber core and Λ represents the grating period. The relationship between the wavelength shift Δ*λ*_B_ of the fiber grating and temperature variation Δ*T* is given by Equation (9).

(9) $$\frac{\Delta \lambda_{B}}{\lambda_{B}}=(\alpha_{f}+\xi_{f})\Delta T$$

where the coefficient of thermal expansion for the fiber core *α_f_* is typically 5.5 × 10^−7^/°C, and the thermo-optic coefficient of the FBG *ξ_f_* is generally 6.4 × 10^−7^/°C.

For demodulation of the EFPI cavity length *l*, the Fourier transform method may be employed to determine the dominant frequency position *m* of the interference spectrum, followed by calculation using Equation (10).

(10) $$l=\frac{cm}{2Nn\Delta \nu }$$

where Δ*v* denotes the optical frequency sampling interval, *N* represents the number of sampling points, and *c* is the light speed.

Based on Equations (3), (4), and (9), the variations in EFPI cavity length and FBG center wavelength in the proposed sensor under combined pressure and temperature loading are expressed as Equation (11).

(11) $$\left[\begin{array}{c} \Delta l\\ \Delta \lambda_{B} \end{array}\right]=\left[\begin{array}{cc} \frac{Lr_{i}^{2}\left(1-2\mu \right)}{E\left(r_{o}^{2}-r_{i}^{2}\right)} & \left(\alpha_{v}-\alpha_{f}\right)L_{a}\\ 0 & \lambda_{B}\left(\alpha_{f}+\xi_{f}\right) \end{array}\right]\left[\begin{array}{c} \Delta P\\ \Delta T \end{array}\right]$$

To simplify the above matrix expression, the parameter calculations are defined as shown in Equation (12).

(12) $$\left\{\begin{array}{c} K_{EFPI}^{P}=\frac{Lr_{i}^{2}\left(1-2\mu \right)}{E\left(r_{o}^{2}-r_{i}^{2}\right)}\\ K_{EFPI}^{T}=\left(\alpha_{v}-\alpha_{f}\right)L_{a}\\ K_{FBG}^{T}=\lambda_{B}\left(\alpha_{f}+\xi_{f}\right) \end{array}\right.$$

It is evident that parameter $K_{EFPI}^{P}\;$ corresponds to the pressure sensitivity of the EFPI cavity, $K_{EFPI}^{T}\;$ represents the temperature sensitivity of the EFPI cavity, and $K_{FBG}^{T}$ denotes the temperature sensitivity of the FBG. Substituting Equation (12) into Equation (11) and solving Equation (11) yields the expression as Equation (13).

(13) $$\left[\begin{array}{c} \Delta P\\ \Delta T \end{array}\right]=\left[\begin{array}{cc} \frac{1}{K_{EFPI}^{P}} & -\frac{K_{EFPI}^{P}}{K_{EFPI}^{P}K_{FBG}^{T}}\\ 0 & \frac{1}{K_{FBG}^{T}} \end{array}\right]\left[\begin{array}{c} \Delta l\\ \Delta \lambda_{B} \end{array}\right]$$

Consequently, once the geometric and physical parameters of the sensor are determined, environmental pressure and temperature can be detected by monitoring variations in the EFPI cavity length and the center wavelength of the FBG.

### 2.3. Sensor Fabrication and Packing

**Figure 4:**
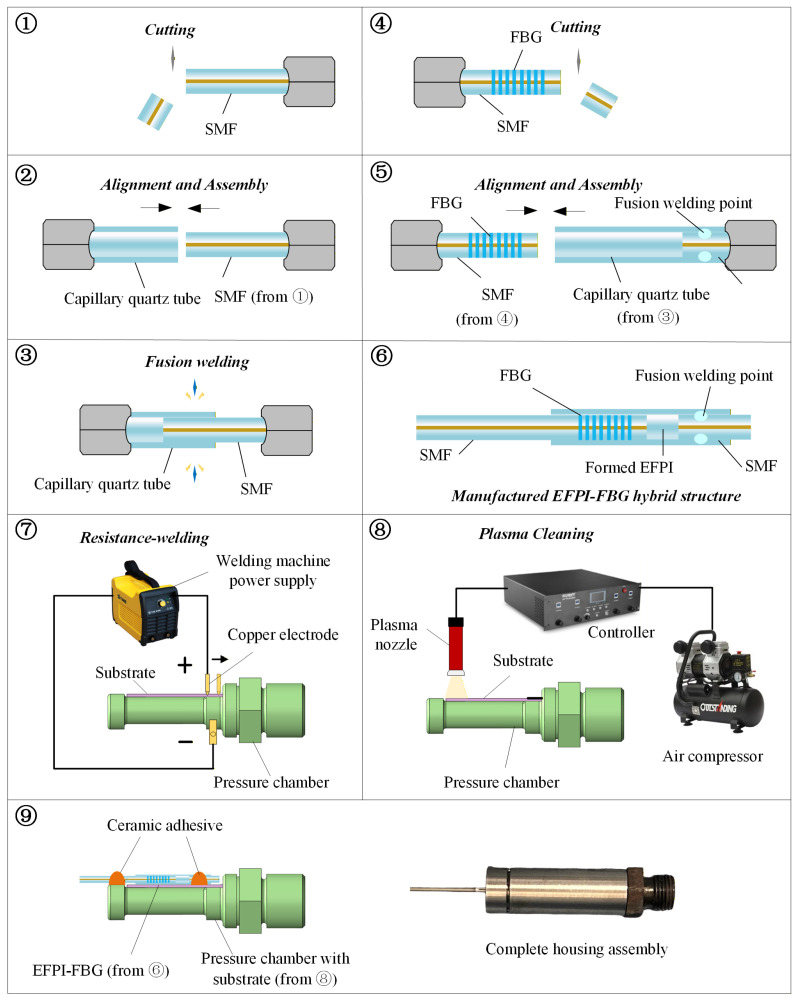
Fabrication and packing sequence of the sensor.

To validate the theoretical design, a sensor prototype for experimental testing was fabricated. Metallic components including the cylindrical pressure chamber, metal substrate, and end cover were obtained through precision machining. The sensor fabrication and packaging sequence is illustrated in Figure 4. A segment of single-mode fiber was cleaved to obtain an optically smooth end-face (step 1). Using a optical fiber welding machine (FITEL185LDF, Furukawa Corporation, Tokyo, Japan), the cleaved fiber was automatically aligned with a capillary quartz tube via machine vision and manually assembled to a predetermined insertion depth (step 2), followed by arc fusion splicing to permanently fix the fiber within the capillary (step 3). The second single-mode fiber containing an FBG was similarly cleaved (step 4) and inserted into the opposite end of the capillary quartz tube, though left unrestrained at this stage (step 5). This configuration established the core EFPI-FBG hybrid sensing structure (step 6). The cylindrical pressure chamber (elastic pressure transducer) was then prepared. To ensure efficient strain transfer to the EFPI-FBG structure: (1) The metal substrate was resistance-welded (DN-5, Hanzhili Co., Ltd., Shanghai, China) to the lateral surface of the pressure chamber (step 7); (2) Plasma cleaning (CRF-APO-RD50-HD, ShenZhen Sing Fung Intelligent Manufacturing Co., Ltd., Shenzhen, China) was applied to the surfaces of both the metal substrate and pressure chamber to enhance adhesion of the high-temperature ceramic adhesive (step 8); (3) The EFPI-FBG structure was finally secured to the surfaces of the pressure chamber and metal substrate using a two-point fixation method with high-temperature inorganic adhesive (step 9). Finally, the housing and ancillary components were installed to complete the sensor prototype.

The geometric and mechanical parameters of the sensor are listed in Table 1. Substituting these parameters into Equation (12) yields the temperature sensitivity and pressure sensitivity of the EFPI, as well as the temperature sensitivity of the FBG within the sensor.

## 3. Experiments, Results, and Discussion

### 3.1. Pressure Experiment

(1) Experimental Setup

**Figure 5:**
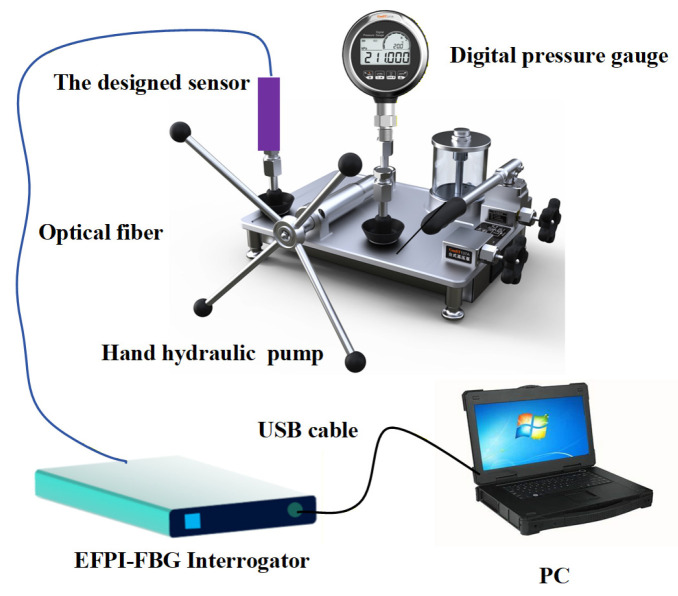
Pressure calibration experimental setup.

**Figure 6:**
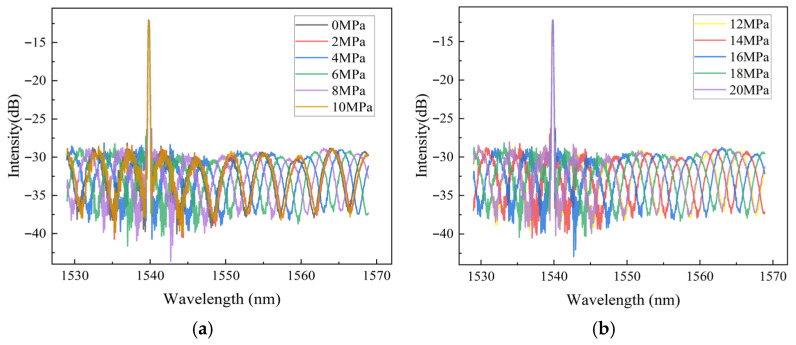
Reflection spectra of the designed sensor under various pressure loadings. (**a**) Reflected spectrum under 0, 2, 4, 6, 8, 10 Mpa. (**b**) Reflected spectrum under 12, 14, 16, 18, 20 Mpa.

To investigate the pressure-sensing characteristics of the proposed sensor, a pressure calibration experiment was conducted under static conditions. As illustrated in Figure 5, the experimental setup mainly comprised a manual hydraulic pump (ConST 137A; pressure range: 0~280 MPa, Beijing Const Instruments Technology Inc., Beijing, China), a digital pressure gauge (ConST 211A; measurement range: 0~100 MPa; accuracy: 0.05%F.S., Beijing Const Instruments Technology Inc., Beijing, China), an EFPI-FBG interrogator (laboratory-developed), and a PC with demodulation software (Version 1, laboratory-developed). The sensor was connected to the hydraulic pump outlet. Broadband light from the demodulator’s integrated source was launched into the sensor, with the reflected signals demodulated upon return. During testing, pressure was incrementally increased from 0 MPa to 20 MPa and then decreased to 0 MPa in 2 MPa steps for 3 times. Each pressure level was maintained for 5 min to ensure stabilization before recording the reflection spectra, EFPI cavity length, and FBG center wavelength via the demodulation software. Under various pressure loadings, the composite reflection spectra of the sensor are shown in Figure 6. It can be observed that as pressure increases, both the period and phase of the EFPI interference spectrum change, with the phase shift being particularly pronounced. This indicates that the specific EFPI structure inside the sensor is highly sensitive to pressure. On the other hand, the central wavelength of the narrowband reflection spectrum of the FBG shows almost no shift with increasing pressure, meaning the FBG structure inside the sensor is insensitive to pressure.

(2) Results and discussion

**Figure 7:**
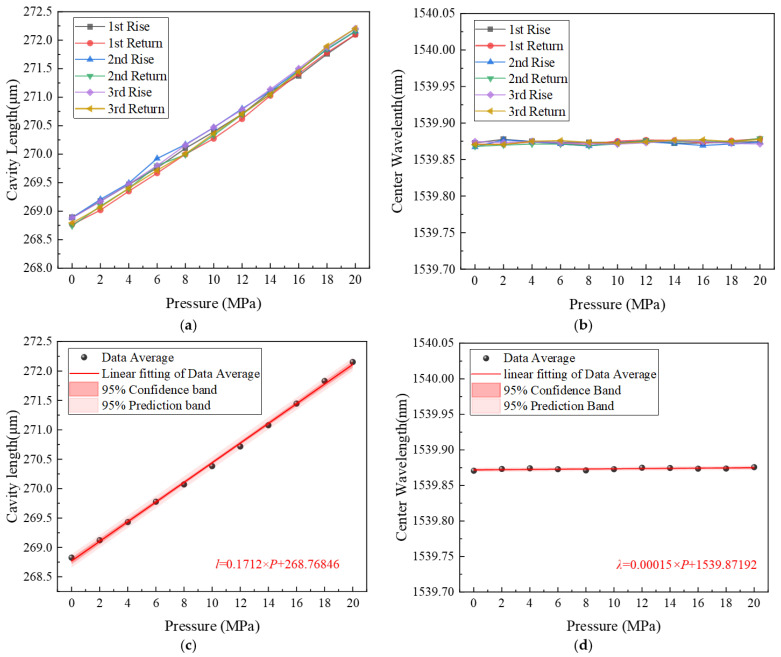
(**a**) Repeated cycle tests of EFPI cavity length vs. pressure. (**b**) Repeated cycle tests of FBG center wavelength vs. pressure. (**c**) Linear fitting curve of EFPI cavity length vs. Pressure. (**d**) Linear fitting curve of FBG center wavelength vs. pressure.

As shown in Figure 7a,b, the test data of EFPI and FBG from three loading-unloading cycles exhibit good repeatability. As the pressure inside the cylindrical elastomer of the sensor increases, the cylinder elongates, leading to an increase in the distance between the two reflective surfaces of the EFPI sensitive element, i.e., an increase in the EFPI cavity length. In contrast, the FBG in the sensor is fixed at one end while the other end is freely suspended within a capillary quartz tube. Therefore, the elongation of the cylinder does not induce stress changes in the FBG structure. When the pressure load varies from 0 to 20 MPa, the cavity length of the EFPI changes by approximately 3 μm, while the central wavelength of the FBG remains essentially stable at 1539.872 nm with no observable variation. The hysteresis error of the EFPI cavity length with respect to pressure is 5.2%F.S., and its repeatability error is 2.8%FS. Figure 7c indicates that within the pressure range of 0~20 MPa, the EFPI cavity length of the designed sensor exhibits a linear variation with pressure. The pressure fitting curve of the EFPI is l = 0.1712×P + 268.76846. The linear correlation coefficient is 0.9986, and the pressure sensitivity is 0.171 μm/MPa. Figure 7d shows the linear fitting results of the FBG central wavelength versus pressure. The fitting equation is l = 0.00015×P + 1539.87192, with a linear correlation coefficient of 0.9402. Its pressure sensitivity is 0.15 pm/MPa, indicating that it is essentially insensitive to pressure changes.

**Figure 8:**
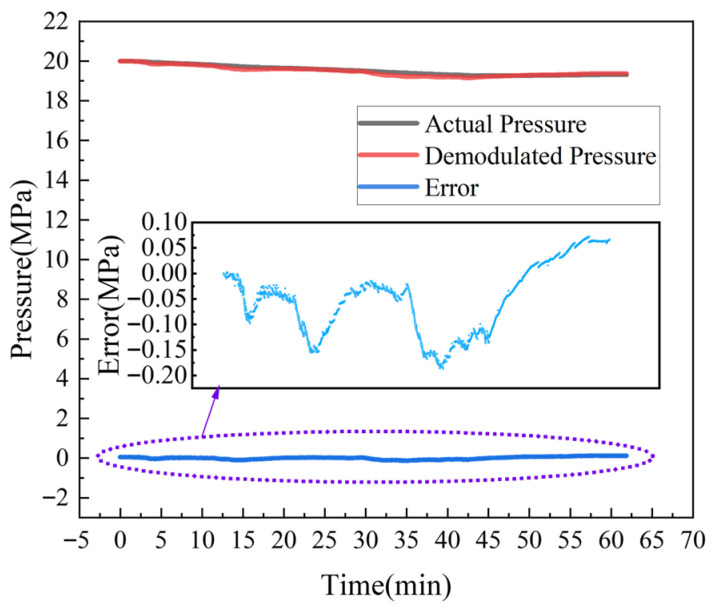
Results from the one-hour pressure holding test at 20 MPa.

To evaluate the stability of the sensor structure under high-pressure conditions, the same pressure loading device in Figure 5 was used to apply and maintain a pressure of 20 MPa on the designed sensor for one hour. A comparison between the test results of the designed sensor and those of the standard pressure sensor (ConST 211A; measurement range: 0~100 MPa; accuracy: 0.05%F.S., Beijing Const Instruments Technology Inc., Beijing, China) is presented in Figure 8. Over time, a certain degree of pressure leakage inevitably occurred in the loading system. Nevertheless, the results from the designed sensor remained consistent with those from the standard pressure sensor throughout the test. During the one-hour pressure-holding test at 20 MPa, the deviation between the two sets of measurements ranged from −0.15 MPa to +0.05 MPa, confirming that the designed sensor exhibits good stability under high-pressure testing.

### 3.2. Temperature Experiment

(1) Experimental setup

**Figure 9:**
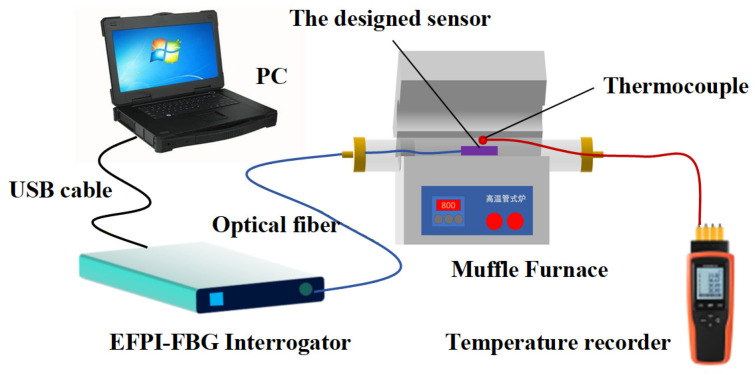
Temperature calibration experimental setup.

**Figure 10:**
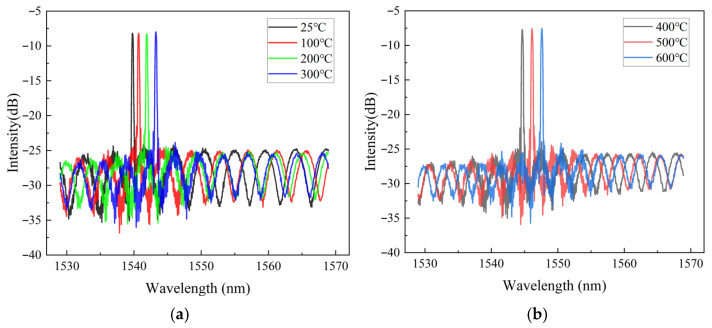
Reflection spectra of the designed sensor under various temperature loadings. (**a**) Reflected spectrum under 25, 100, 200, 300 °C. (**b**) Reflected spectrum under 400, 500, 600 °C.

To investigate the temperature-sensing characteristics of the designed sensor, a temperature calibration experiment was conducted under static conditions. As shown in Figure 9, the experimental setup mainly comprised an EFPI-FBG interrogator, a PC with demodulation software, and a muffle furnace (XS1200, operating range: RT-1200 °C, Hefei Kejing Materials Technology Co., Ltd., Hefei, China). Additionally, a high-accuracy thermocouple temperature sensor (YET-640X; range: −40 °C to 900 °C; accuracy: ±[0.3% rdg + 0.4 °C], Shenzhen Yuwen Measurement Technology Co., Ltd., Shenzhen, China) served as the reference temperature standard. During testing, both the thermocouple and the designed sensor were placed at identical locations within the temperature-controlled muffle furnace. Under various temperature loadings, the composite reflection spectra of the sensor are shown in Figure 10. As temperature rises, changes in both the period and phase of the EFPI interference spectrum are evident, with the phase shift again being particularly noticeable, demonstrating the temperature-sensitive nature of the EFPI structure. In contrast, the central wavelength of the FBG’s narrowband reflection spectrum exhibits a significant rightward shift with increasing temperature, indicating that the FBG structure is highly sensitive to temperature. This study leverages the distinct sensitivities of EFPI and FBG fiber sensing elements to temperature and pressure to achieve simultaneous measurement of both parameters.

(2) Results and discussion

**Figure 11:**
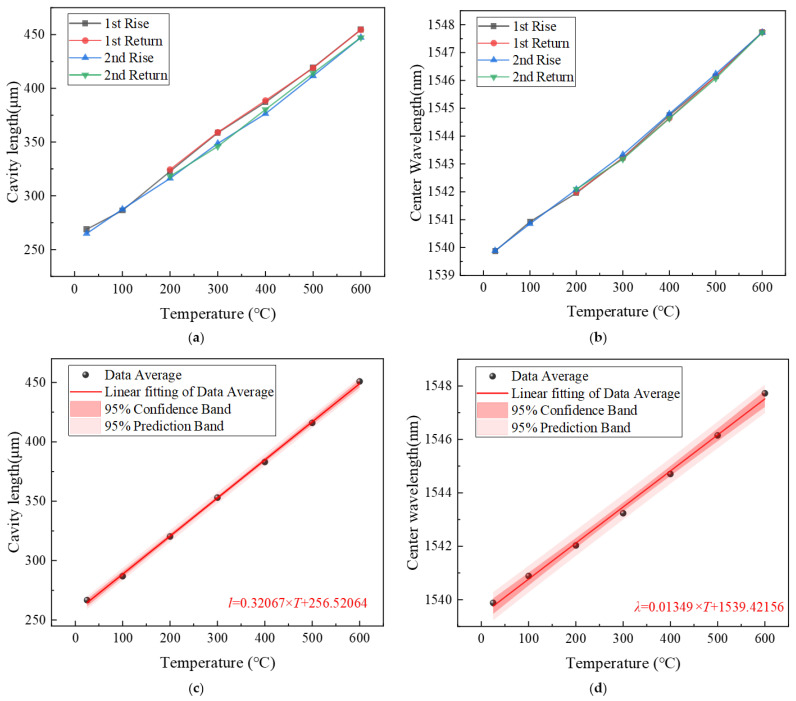
(**a**) Repeated cycle tests of EFPI cavity length vs. temperature. (**b**) Repeated cycle tests of FBG center wavelength vs. temperature. (**c**) Linear fitting curve of EFPI cavity length vs. Temperature. (**d**) Linear fitting curve of FBG center wavelength vs. temperature.

The experimental results in Figure 11 show that within the temperature range of 25 °C to 600 °C, both the EFPI cavity length and the FBG central wavelength of the designed fiber-optic sensor exhibit a linear variation with temperature. To evaluate the hysteresis error and repeatability error of the sensor, the ambient temperature was set at 25, 100, and 200 °C. Then, the temperature was increased from 200 °C to 600 °C in steps of 100 °C, followed by a return to 200 °C, another increase to 600 °C, and finally a return to 200 °C. As shown in Figure 11a, the hysteresis error of the EFPI cavity length relative to temperature changes is 2.12%F.S., and the repeatability error is 4.04%F.S. According to Figure 11b, the hysteresis error of the FBG center wavelength relative to temperature changes is 1.69%F.S., and the repeatability error is 3.15%F.S. Figure 11c,d present the linear fitting results of the EFPI cavity length and FBG center wavelength relative to temperature changes, respectively. The temperature fitting curve of the EFPI is l = 0.236×T + 262.218. The linear correlation coefficient is 0.9994, and the temperature sensitivity is 0.321 μm/°C. The temperature fitting curve of the FBG is $\lambda_{B}\;=\;0.0137\;\times T+1539.326$. The linear correlation coefficient is 0.9970, and the temperature sensitivity is 13.5 pm/°C.

The actual relationship between the optical fiber EFPI-FBG temperature/pressure composite sensor and the external environmental parameters can be expressed as follows:

$$\left[\begin{array}{c} \Delta \lambda_{B}\left(\mathrm{pm}\right)\\ \Delta l\left(\mu m\right) \end{array}\right]=\left[\begin{array}{cc} 0 & 13.5\\ 0.171 & 0.321 \end{array}\right]\left[\begin{array}{c} \Delta P\;\left(\mathrm{MPa}\right)\\ \Delta T\;\left({}^{\circ}C\right) \end{array}\right]$$

**Figure 12:**
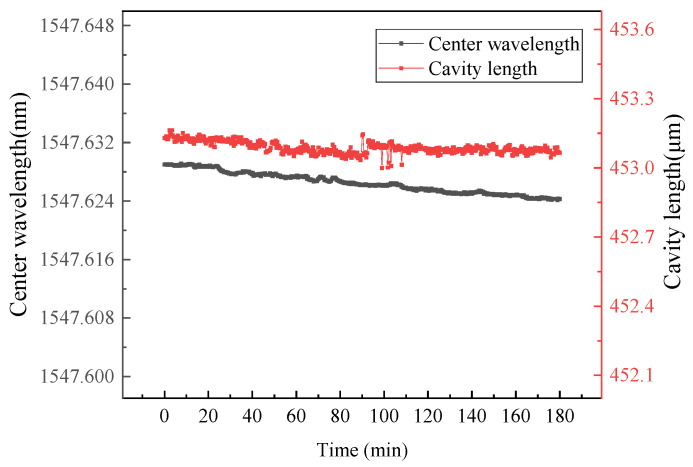
Results from the three-hour temperature holding test at 600 °C.

To verify the long-term stability of the designed and fabricated sensor in high-temperature environments, the sensor was placed in a tube furnace as shown in Figure 9 and maintained at 600 °C for three hours (thermocouple measurements indicated that the temperature fluctuation of the furnace at 600 °C was 0.5 °C). The EFPI cavity length and FBG center wavelength of the sensor were continuously recorded. Figure 12 presents the long-term high-temperature test results of the sensor. Over the three-hour period at 600 °C, the drift of the EFPI cavity length and the FBG center wavelength was approximately 0.2 μm and 5 pm, respectively. This demonstrates the excellent high-temperature stability of the sensor (Table 2).

## 4. Conclusions

This study presents a composite sensor structure based on EFPI-FBG, establishes its fabrication process and packaging methodology, and develops a temperature-pressure decoupling matrix. Experimental results demonstrate that the proposed optical fiber EFPI-FBG composite sensor withstands high temperatures up to 600 °C. Within the pressure range of 0~20 MPa, it achieves a pressure sensitivity of 0.171 μm/MPa. Over the temperature range of 25~600 °C, both the EFPI cavity length and the FBG central wavelength vary linearly with temperature, showing sensitivities of 0.321 μm/°C and 13.5 pm/°C, respectively. By applying a decoupling matrix, simultaneous measurement of temperature and pressure is realized. The proposed sensor provides a new technical approach and an effective solution for pressure measurement in high-temperature environments. It should be noted that the current work has certain limitations: (1) Due to the presence of cantilever structures within the sensor, high-frequency pressure impacts may excite its vibration modes. Therefore, this sensor is primarily suited for static or quasi-static pressure testing. (2) The decoupling matrix was established based on sensitivities obtained at room temperature (for pressure) and under zero pressure (for temperature). Due to constraints in the experimental setup, simultaneous and precise control of high pressure (up to 20 MPa) and high temperature (up to 600 °C) was not feasible. Future work will focus on constructing a dedicated high-temperature, high-pressure calibration platform to systematically investigate these coupling effects and develop a more comprehensive and accurate decoupling algorithm.

## Figures and Tables

**Table 1:** Dimensions and mechanical properties of theoretical model.

Symbol	Parameter Identity of the Cylinder	Value	Unit
*r_i_*	inner radius	2	mm
*r_o_*	outer radius	2.4	mm
*E*	Young’s modulus	200	GPa
*μ*	Poisson’s ratio	0.3	/
*α_v_*	coefficient of thermal expansion	$18.9\;\times \;10^{-6}$	°C^−1^
*L*	initial effective length	21	mm
*L_a_*	Two-point bonding distance of the EFPI-FBG	11	mm

**Table 2:** Performance comparison of the sensors.

Reference	Structure	Pressure Sensitivity	Pressure Range	Temperature Sensitivity	Temperature Range
[25]	Single EFPI	3.54 μm/MPa	0–1 MPa	/	23–800 °C
[27]	Double FPI	1.80 μm/MPa	0–5 MPa	3300 pm/°C	20–1400 °C
[30]	Double FBG	50.6 ×10^−6^ μm/MPa	0–40 MPa	31.4 pm/°C	50–200 °C
This work	EFPI-FBG	0.171 μm/MPa	0–20 MPa	13.5 pm/°C	25–600 °C

## Data Availability

The data presented in this study are available on request from the corresponding author due to legitimate research purposes.
